# Sustainability in Asphalt, Concrete and Other Pavement Materials: Design, Performance, and Characterization

**DOI:** 10.3390/ma19091890

**Published:** 2026-05-04

**Authors:** Wentao Wang, Linbing Wang

**Affiliations:** 1 National Center for Materials Service Safety, University of Science and Technology Beijing, Beijing 100083, China; wentaowang@ustb.edu.cn; 2 The Sensing and Perception Lab, School of Environmental, Civil, Agricultural and Mechanical Engineering, University of Georgia, Athens, GA 30602, USA

## 1. Background and Scope

With the continuous growth of global infrastructure construction and increasing environmental pressures [1,2,3,4], the field of road engineering and construction materials is undergoing a profound green transformation. Asphalt, concrete, and various pavement materials consume substantial natural resources and emit significant greenhouse gases during production, construction, and service [5,6,7]. There is an urgent need to promote sustainable technologies through material design, performance evaluation, and functional characterization [8,9,10]. This Special Issue, “Sustainability in Asphalt, Concrete and Pavement Materials: Design, Performance, and Characterization,” aims to present the latest research findings in sustainable pavement materials, covering areas such as material modification, waste valorization, low-carbon cementitious systems, performance prediction, and advanced characterization methods.

A total of 20 high-quality papers, all rigorously peer-reviewed, are included in this Special Issue. The research topics involve warm-mix asphalt technology, the recycling of cigarette butts for fiber reuse, the materialization of industrial/agricultural by-products such as steel slag and olive ash, the evaluation of strength and durability of cement-based materials, and advanced characterization techniques. These studies not only demonstrate the important role of materials science in sustainable transportation infrastructure but also provide a scientific basis for engineering design, standard formulation, and policy orientation.

## 2. Overview of Research Progress

Based on an analysis of all 20 papers published in this Special Issue, the research can be broadly categorized into the following key thematic areas.

(1) **Microwave Heating and Curing Technologies for Asphalt Mixtures**: Xu et al. [Contribution 1] and Jiang et al. [Contribution 2] developed SiC–Fe_3_O_4_ composites to enhance microwave absorption in emulsified asphalt mixtures. The optimal conditions (4% composite with 1:1 SiC–Fe_3_O_4_ ratio, 600–1000 W microwave power) significantly accelerated curing and improved early strength without compromising road performance.
(2) **Sustainable Binders and Supplementary Cementitious Materials**: Belaidi et al. [Contribution 3] systematically investigated the effects of olive pomace ash as a partial cement replacement on workability, mechanical properties, and freeze–thaw durability, identifying 20% as the optimal replacement rate. Wang et al. [Contribution 4] systematically investigated the influence of warm-mix additive dosage on the viscoelastic properties and VOC emissions of SBS-modified asphalt binder. They found that 0.4–0.5% additive can significantly reduce construction temperature and harmful gas emissions without compromising pavement performance. Huang et al. [Contribution 5] demonstrated that slate coarse aggregates exhibit crushing values (9.2%) lower than basalt and limestone, with excellent water and heat resistance, confirming their viability as sustainable alternatives in aggregate-scarce regions.
(3) **Non-Destructive Testing and Performance Characterization**: Wang et al. [Contribution 6] employed 3D laser scanning technology to quantitatively evaluate the evolution of surface roughness in asphalt mixtures under dynamic water pressure, revealing the coupled damage mechanism of fine aggregate matrix (FAM) stripping and aggregate degradation. Hou et al. [Contribution 7] proposed an evidential regression deep network for permittivity measurement in heterogeneous concrete. The method quantified both aleatory and epistemic uncertainties, achieving a mean square error of 7.50% on 1500 samples. Abdulkadir et al. [Contribution 8] systematically compared the reliability of three non-destructive testing methods (ultrasonic pulse velocity, electrical resistivity, and acoustic emission) for strength prediction of cemented paste backfill through a meta-analysis and proposed a multi-parameter fusion prediction model.
(4) **Mechanical Behavior and Durability of Innovative Pavement Materials**: Chen et al. [5] investigated water vapor effects on asphalt concrete fracture resistance. Increasing relative humidity from 2% to 100% reduced tensile strength by 34.8% on average; genetic expression programming models captured the coupled effects of humidity and temperature with R^2^ > 0.94. Jatoi et al. [Contribution 9] compared Marshall and Superpave mix designs. Superpave mixtures exhibited superior rutting resistance, fatigue life, and moisture damage mitigation, especially when combined with polymer-modified binders and rejuvenators. Varol Morova et al. [Contribution 10] used recycled cigarette butt-derived cellulose acetate fibers in SMA mixtures. At 0.3–0.4% fiber content, the recycled fibers achieved comparable performance to commercial cellulose fibers with 6–8% cost savings. Li et al. [Contribution 11] developed polyurethane/graphene oxide (PU/GO) composites for self-healing asphalt. The composite (1.6% GO) doubled PU tensile strength and achieved 100% ductility self-healing after 15 min infrared exposure. Yu et al. [Contribution 12] and Wang et al. [Contribution 13] studied aggregate packing characteristics and force chain networks using the discrete element method. SMA mixtures exhibited superior skeleton structures, and strong correlations (R^2^ > 0.7) were found between packing and mechanical performance. Zhang et al. [Contribution 14] compared activated versus conventional rubber in SBS-modified asphalt. Activated rubber formed a uniform micro particle network, increasing complex modulus by 417.16%. Nie et al. [Contribution 15] and Lv et al. [Contribution 16] explored sustainable concrete formulations using modified sludge soil and aeolian sand, respectively. Both studies demonstrated that locally available waste materials can be engineered to meet structural requirements. Additional contributions addressed seismic response mitigation of railway bridges [Contribution 17], non-destructive testing of existing pavements using geostatistics [Contribution 18], and comparative analysis of steel slag powders in asphalt mastic [Contribution 19].

## 3. Future Outlook

Although this Special Issue covers several important research directions in sustainable materials, several areas warrant further exploration:

(1) **Synergistic Utilization of Multi-Source Solid Wastes**: Future research should focus on the combined effects of various industrial/agricultural solid wastes in pavement materials and develop integrated design methods based on performance, cost, and environmental impact.
(2) **High-Performance Warm and Cold Mix Technologies**: Further reduction in construction temperatures should be pursued while simultaneously enhancing the long-term durability and aging resistance of asphalt mixtures.
(3) **Non-Destructive Testing and Intelligent Monitoring**: Real-time monitoring systems integrating multi-physics signals (ultrasonics, resistivity, acoustics, imaging) should be developed and combined with digital twins and Internet of Things technologies.
(4) **Life Cycle Assessment**: The carbon emissions and resource consumption of sustainable pavement materials should be systematically quantified across raw material acquisition, construction, maintenance, and end-of-life stages to support green certification and carbon trading initiatives.

We believe that the research studies presented in this Special Issue will serve as a valuable reference for researchers, engineers, and policymakers in the fields of road engineering and construction materials, and will contribute to the development of greener, more durable, and intelligent infrastructure.
